# Tissue Inhibitor of Metalloproteinases-1 Overexpression Mediates Chemoresistance in Triple-Negative Breast Cancer Cells

**DOI:** 10.3390/cells12131809

**Published:** 2023-07-07

**Authors:** Lisa Agnello, Annachiara d’Argenio, Alessandra Caliendo, Roberto Nilo, Antonella Zannetti, Monica Fedele, Simona Camorani, Laura Cerchia

**Affiliations:** 1 Institute of Experimental Endocrinology and Oncology “G. Salvatore”, National Research Council (CNR), 80131 Naples, Italy; l.agnello@ieos.cnr.it (L.A.); a.dargenio@ieos.cnr.it (A.d.); alessandracaliendo9@gmail.com (A.C.); r.nilo@ieos.cnr.it (R.N.); mfedele@unina.it (M.F.); s.camorani@ieos.cnr.it (S.C.); 2 Institute of Biostructures and Bioimaging, National Research Council (CNR), 80145 Naples, Italy; antonella.zannetti@ibb.cnr.it

**Keywords:** chemoresistance, triple-negative breast cancer, tumor microenvironment, tissue inhibitor of metalloproteinases-1, CD63 cell receptor

## Abstract

Triple-negative breast cancer (TNBC) is among the most aggressive breast cancer subtypes. Despite being initially responsive to chemotherapy, patients develop drug-resistant and metastatic tumors. Tissue inhibitor of metalloproteinases-1 (TIMP-1) is a secreted protein with a tumor suppressor function due to its anti-proteolytic activity. Nevertheless, evidence indicates that TIMP-1 binds to the CD63 receptor and activates noncanonical oncogenic signaling in several cancers, but its role in mediating TNBC chemoresistance is still largely unexplored. Here, we show that mesenchymal-like TNBC cells express TIMP-1, whose levels are further increased in cells generated to be resistant to cisplatin (Cis-Pt-R) and doxorubicin (Dox-R). Moreover, public dataset analyses indicate that high TIMP-1 levels are associated with a worse prognosis in TNBC subjected to chemotherapy. Knock-down of TIMP-1 in both Cis-Pt-R and Dox-R cells reverses their resistance by inhibiting AKT activation. Consistently, TNBC cells exposed to recombinant TIMP-1 or TIMP-1-enriched media from chemoresistant cells, acquire resistance to both cisplatin and doxorubicin. Importantly, released TIMP-1 reassociates with plasma membrane by binding to CD63 and, in the absence of CD63 expression, TIMP-1-mediated chemoresistance is blocked. Thus, our results identify TIMP-1 as a new biomarker of TNBC chemoresistance and lay the groundwork for evaluating whether blockade of TIMP-1 signal is a viable treatment strategy.

## 1. Introduction

Triple-negative breast cancer (TNBC), representing approximately 15–20% of all breast cancer (BC) diagnosed worldwide, is defined by the lack of estrogen receptor (ER), progesterone receptor (PR) and epidermal growth factor receptor 2 (HER2) [1,2]. TNBC clinical features include high invasiveness, metastatic potential and rate of relapse [3]. Unfortunately, despite having higher rates of clinical response to neoadjuvant chemotherapy, TNBC patients show higher risk of recurrence than women with hormone-positive BC [4,5]. Approximately 50% of TNBC patients develop distant metastases, which occur mostly in the third year after diagnosis and involve the brain and visceral organs, and less than 30% of metastatic TNBC patients survive their cancer beyond 5 years from the diagnosis [3].

Ruling out the possibility of using therapies against ER, PR and HER2, and due to the limited options of targeted therapies that are available only for a restricted group of patients, chemotherapy is the mainstay of systemic treatment for patients with both early and advanced TNBC [6,7,8]. However, after an initial response to chemotherapeutics, TNBC tends to acquire epithelial–mesenchymal transition (EMT) features and a stemness phenotype, which promote metastases and chemoresistance [9].

Among the numerous players of chemoresistance in TNBC, secretory proteins produced by cancer and stromal cells are important components of the tumor microenvironment (TME) with a key role in this process, affecting sensitivity to therapy [10,11].

Tissue inhibitor of metalloproteinases-1 (TIMP-1) is a secreted and soluble glycoprotein belonging to the TIMP family, including members TIMP-1 to TIMP-4, which has the function of inhibiting the proteolytic activity of matrix metalloproteinases (MMPs) and other metalloproteinases within the extracellular matrix (ECM) [12].

TIMP-1 consists of 184 amino acids organized into two structural domains, N-terminal and C-terminal, each containing three disulfide bonds. The N-terminal domain is involved in the inhibition of the proteolytic activity of metalloproteinases by interacting with the MMP catalytic site to form a 1:1 stoichiometric noncovalent complex [13,14]. Although it has long been recognized as a tumor suppressor protein due to its inhibitory role on MMPs, it is now clear that TIMP-1, independently of its inhibitory activity on MMPs, can activate several intracellular signaling pathways involved in cell growth, proliferation, differentiation, stemness and EMT, exerting an oncogenic activity [15,16,17]. Accordingly, increasing evidence demonstrates that TIMP-1 is overexpressed in several types of human cancers, including lung cancer [18,19], colon cancer [20], prostate cancer [21], melanoma [22], glioblastoma [23] and breast cancer [24,25], and its increased expression is correlated with a poor prognosis of the patient.

In 2006, the tetraspanin CD63 was identified as the cell surface receptor partner responsible for mediating TIMP-1 oncogenic activity [26]. By using deletion mutants of TIMP-1, the structure–function relationship of TIMP-1 for its interaction with CD63 was elucidated in detail, demonstrating that, while the N-terminal domain of TIMP-1 is sufficient for TIMP-1 inhibitory activity on MMPs, nine C-terminal amino acid residues are required for binding to CD63, thus eliciting pro-tumorigenic function [27]. Several studies have proven that either TIMP-1 overexpression in human non-malignant mammary epithelial MCF10A cells or cell treatment with exogenously added recombinant TIMP-1 confers tumorigenic features, including inhibition of apoptosis [26,28,29,30], EMT and increased cell motility [31], through interaction of TIMP-1 with CD63 on the cell surface. Moreover, TIMP-1 expression increases in MCF10A cells as the cells become more aggressive following H-ras transfection [32]. In the presence of TIMP-1, CD63 can interact with integrin β1, forming a TIMP-1/CD63/integrin β1 complex, which leads to malignant progression, as in the case of melanoma genesis [33]. More recently, it has been shown that TIMP-1 secreted by carcinoma-associated fibroblasts stimulates breast cancer cell-dependent secretion of TIMP-1, which in turn cooperates with CD63 and integrin β1 to promote tumor cell growth and migration [34].

The analysis of TIMP-1 mRNA expression in breast cancer specimens revealed that TIMP-1 is expressed more highly in TNBC than in normal breast tissue, and the higher serum TIMP-1 levels in the malignant tissue are associated with a poor prognosis. Furthermore, blocking TIMP-1 by neutralizing antibodies inhibited the growth of implanted TNBC in mice, suggesting TIMP-1 as an actionable biomarker for this tumor [25].

Even if some studies have documented a role for TIMP-1 in chemoresistance [35,36,37], its involvement as a mediator of chemoresistance in TNBC cells has never been explored.

To fill this knowledge gap, by employing doxorubicin (Dox) and cisplatin (Cis-Pt), two chemotherapeutics used in clinic for TNBC, we generated chemoresistant TNBC MDA-MB-231 cell lines (Dox-R and Cis-Pt-R) characterized by a chemoresistant mesenchymal phenotype and TIMP-1 overexpression. We report a critical role for TIMP-1, and its binding partner CD63, in this chemoresistance.

## 2. Materials and Methods

### 2.1. Cell Lines and Culture Conditions

Human TNBC MDA-MB-231 and BT-549, triple-positive breast cancer (TPBC) BT-474 and mammary epithelial MCF10A cell lines were purchased from the American Type Culture Collection (ATCC, Manassas, VA, USA). MDA-MB-231 and BT-549 were grown in Roswell Park Memorial Institute-1640 medium (RPMI-1640, Sigma-Aldrich, Milan, Italy) supplemented with 10% fetal bovine serum (FBS, Sigma-Aldrich); BT-474 were grown in Hybri-Care medium (ATCC) supplemented with 10% FBS; MCF10A were grown in Dulbecco’s Modified Eagle’s Medium/Ham’s Nutrient Mixture F-12 (DMEM/F-12, Sigma-Aldrich) supplemented with 5% horse serum (ThermoFisher Scientific, Waltham, MA, USA), 0.5 µg/mL hydrocortisone, 20 ng/mL epidermal growth factor, 10 ng/mL cholera toxin and 10 µg/mL insulin (Sigma-Aldrich). All cells were maintained in 95% air/5% CO_2_ atmosphere at 37 °C.

MDA-MB-231 cells chronically resistant to Dox (Dox-R) or Cis-Pt (Cis-Pt-R) were generated as previously reported [38,39]. Briefly, MDA-MB-231 cells were exposed to an initial concentration of 6.6 µM Cis-Pt or 0.105 µM Dox in RPMI-1640 plus 10% FBS for 24 h. Subsequently, the cells were washed three times with Dulbecco’s phosphate-buffered saline (DPBS, Sigma-Aldrich), trypsinized and split. The cell population that survived was grown to 80% confluency and, to ensure viability, was passaged once a week over a period of at least two weeks. The surviving population of cells was exposed to a drug concentration increased to 13 µM, 18 µM, 20 µM and 25 µM (Cis-Pt), or 200 nM, 300 nM and 400 nM (Dox). Dox-R and Cis-Pt-R were kept in culture with a fixed concentration of 5 nM Dox and 1 µM Cis-Pt, respectively.

### 2.2. Immunoblot

Cell lysate preparation and immunoblot analyses were performed as previously reported [40]. Filters were probed with the indicated primary antibodies: anti-TIMP-1 and anti-CD99 (R&D Systems, Minneapolis, MN, USA); anti-integrin β1, anti-CD63 and anti-vinculin (Santa Cruz Biotechnology, Dallas, TX, USA); anti-vimentin, anti-integrin αv, anti-PDGFRβ, anti-pAKT, anti-pErk 1/2 and anti-ZO-1 (Cell Signaling Technology Inc., Danvers, MA, USA); anti-ITPRIPL1 (OriGene Technologies, Rockville, MD, USA); anti-NF-kB-p65 (Elabscience, Houston, TX, USA); anti-actin (Sigma-Aldrich); and anti-CD44 (Abcam, Cambridge, UK). Densitometric analysis was performed on at least two different exposures to assure the linearity of each acquisition using ImageJ software (v1.46r).

### 2.3. Public Datasets, Data Mining and Analysis

For mRNA expression analysis in human specimens and correlation with clinical, molecular and cell phenotypes, the Genomics Analysis and Visualization platform (http://r2.amc.nl) and Kaplan–Meier plotter webtool (www.kmplot.com) were used with the following datasets from the Gene Expression Omnibus (GEO) database and The Cancer Genome Atlas (TCGA) program: GSE76124 [41], including 114 basal-like, 37 luminal androgen receptor and 47 mesenchymal TNBCs; a TCGA gene chip mRNA dataset, including 186 patients with TNBC at third histologic grade, and only treated with chemotherapeutics [42].

### 2.4. Cell Transfection

Chemoresistant (Cis-Pt-R and Dox-R) and parental MDA-MB-231 cells (1.8 × 10^5^ cells, 6-well plates) were transfected for 5 h at 37 °C with 66 nM TIMP-1-targeting siRNA (si-TIMP-1) and CD63-targeting siRNA (si-CD63) (IDT, Coralville, IA, USA), respectively, by using Lipofectamine RNAiMAX Reagent (ThermoFisher Scientific), according to the manufacturer’s instructions. Scrambled non-targeting siRNA (siRNA ctrl) was used as a negative control (Qiagen, Hilden, Germany).

Sequences of si-TIMP-1 were: sense sequence, 5′-GCACAGUGUUUCCCUGUUUAUTT-3′ and anti-sense sequence, 5′-AAAUAAACAGGGAAACACUGUGCTT-3′.

A mixture of three different si-CD63 sequences were used: CD63 1, sense sequence, 5′-GGUGGAAGGAGGAAUGAAATT-3′ and anti-sense sequence, 5′-UUUCAUUCCUCCUUCCACCTT-3′; CD63 2, sense sequence, 5′-GGCAGCAGAUGGAGAAUUATT-3′ and anti-sense sequence, 5′-UAAUUCUCCUCUGCUGCCTT-3′; and CD63 3, sense sequence, 5′-GUGGCUACGAGGUGAUGUATT-3′ and anti-sense sequence, 5′-UACAUCACCUCGUAGCCACTT-3′. At 24 h and 48 h post-transfection with si-TIMP-1 and si-CD63, respectively, cell lysates were analyzed by immunoblotting.

### 2.5. Cell Viability Assay

For EC50 value calculation, parental MDA-MB-231 cells and Dox-R and Cis-Pt-R cells untransfected, or 24 h after transfection with si-TIMP-1 or siRNA ctrl (5000 cells/well, 96-well plates), were treated with increasing doses of Dox (ranging from 10 nM to 100 µM) or Cis-Pt (ranging from 100 nM to 200 µM). Viability of cells following 48 h treatment was assessed with Thiazolyl Blue Tetrazolium Bromide (MTT, AppliChem GmbH, Darmstadt, Germany), according to the manufacturer’s protocol, and expressed as percent of viable treated cells with respect to untreated cells. Data about cell viability were plotted in GraphPad Prism v.8.4.3 to draw a dose–response curve and to determine the EC50.

For assessing the effect of exogeneous TIMP-1 on cell viability, MDA-MB-231 (5000 cells/well, 96-well plates) and MDA-MB-231 cells interfered for CD63 expression (7000 cells/well, 96-well plates) were left untreated or treated for 48 h with 1 μM Dox or 20 µM Cis-Pt alone or in combination with either recombinant TIMP-1 (25 ng/mL) (Biotechne, Minneapolis, MN, USA) or TIMP-1 enriched conditioned media (CM) obtained from Dox-R cells. Cis-Pt-R and Dox-R cells treated with 20 µM Cis-Pt or 1 μM Dox, respectively, were used as chemoresistant reference cells. Cell viability was evaluated as described above. To assess the effect of the PI3K/AKT pathway on TIMP-1-mediated chemoresistance, MDA-MB-231 cells were treated with recombinant TIMP-1 plus Cis-Pt or Dox, as described above, in the presence or absence of 10 μM LY294002 (Thermo Fisher Scientific).

### 2.6. Cell Apoptosis by Flow Cytometry Analysis

After 24 h transfection with si-TIMP-1 or siRNA ctrl, Dox-R and Cis-Pt-R cells were detached from culture plates with 0.02% EDTA (Invitrogen, Waltham, MA, USA) and 1 × 10^6^ cells were stained with Annexin-V and propidium iodide (PI) by using Annexin-V-FLUOS staining Kit (Roche Diagnostics GmbH, Mannheim, Germany), as previously reported [43]. Cells were analyzed via flow cytometry, measuring the fluorescence emission at 530 nm (Annexin-V) and 615 nm (PI) using BD AccuriTM C6.

### 2.7. Conditioned Medium Preparation

MDA-MB-231, BT-549, Dox-R, Cis-Pt-R, BT-474 and MCF10A cells (1 × 10^6^ cells, 100 mm plates) were plated in their proper culture medium supplemented with serum. After 72 h, the media were collected from 3 plates for each cell line and concentrated with an Amicon Ultra 4-kDa centrifugal filter device (Merck Millipore). An equal volume of each medium was analyzed for TIMP-1 expression by immunoblotting.

### 2.8. Membrane Protein Preparation

Membrane and cytoplasmic proteins from 2 × 10^6^ MDA-MB-231, Dox-R, Cis-Pt-R and BT-549 cells were isolated using a Subcellular Protein Fractionation Kit for Cultured Cells (Thermo Scientific, Waltham, MA, USA), according to the provider’s instructions. An equal volume of each fraction was analyzed via immunoblotting.

### 2.9. Confocal Microscopy

MDA-MB-231, BT-549, Cis-Pt-R and Dox-R (1.0 × 10^5^ cells/well, 24-well plates) were seeded on a coverslip for 24 h and then fixed in 4% paraformaldehyde/DPBS for 10 min at room temperature (RT). For CD63 staining, cells were subjected to blocking in 3% FBS for 1 h at RT and then incubated with anti-CD63 antibody (Santa Cruz Biotechnology), washed three times in DPBS and incubated with Fluor 488 anti-mouse (Invitrogen). For dual staining of CD63 and TIMP-1, cells were subjected to blocking in 2% bovine serum albumin for 1 h at RT and then incubated with anti-CD63 (Proteintech, Rosemont, IL, USA) and anti-TIMP-1 (ThermoFisher Scientific) antibodies, followed by three washes with DPBS and incubation with Alexa Fluor 488 anti-mouse (anti-TIMP-1) and Alexa Fluor 567 anti-rabbit (anti-CD63). Finally, after three washes in DPBS, cells were incubated with 1.5 μM 4′,6-Diamidino-2-phenylindole (DAPI, Sigma-Aldrich) and mounted with glycerol/DPBS. Samples were visualized by Zeiss LSM 700 META confocal microscopy equipped with a Plan-Apochromat 63×/1.4 Oil DIC objective.

### 2.10. Immunoprecipitation

One mg Cis-Pt-R cell lysate was incubated with 4 μg anti-CD63 (Santa Cruz Biotechnology) or anti-integrin β1 (R&D Systems) antibodies. After 2 h incubation at 4 °C, immunoprecipitation was performed with protein A/G-agarose (Santa Cruz Biotechnology Inc.) overnight at 4 °C.

### 2.11. Statistical Analysis

All statistical values were defined using GraphPad Prism version 8.4.3 with Student’s *t*-test (two variables) or one-way analysis of variance (ANOVA) followed by Tukey’s multiple comparison test (more than two variables). *p* value < 0.05 was considered significant for all analyses.

## 3. Results

### 3.1. TIMP-1 Expression Is Upregulated in Chemoresistant TNBC Cells

**Figure 1:**
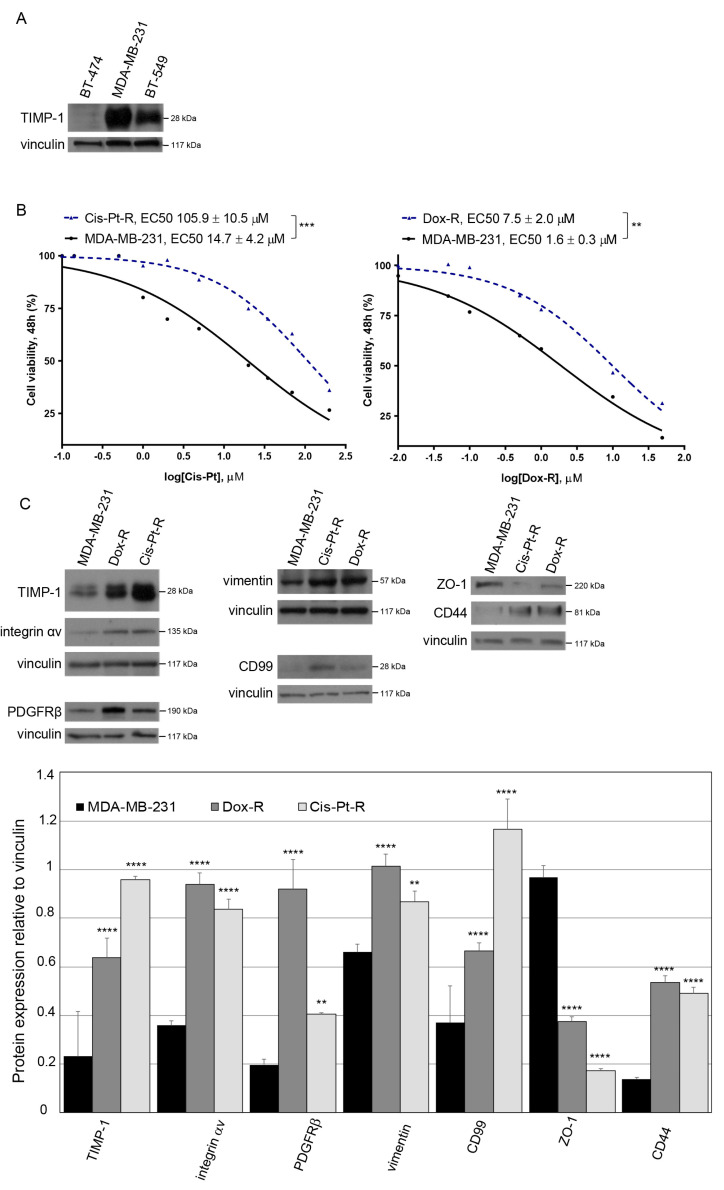
TIMP-1 expression is upregulated in chemoresistant TNBC cell lines. (**A**) Lysates from TNBC MDA-MB-231 and BT-549 cells and TPBC BT-474 cells were immunoblotted with anti-TIMP-1 antibody. Equal loading was confirmed by immunoblot with anti-vinculin antibody. (**B**) Cis-Pt-R and Dox-R cells exhibit increased fold changes in EC50 with respect to parental MDA-MB-231 cells. Following 48 h of Dox or Cis-Pt treatment, cell viability was determined and expressed as percentage of viable treated cells with respect to untreated controls. EC50 was estimated on the basis of three independent experiments. ** *p* < 0.01, *** *p* < 0.001; unpaired *t*-test. (**C**) Lysates from parental and chemoresistant cell lines were immunoblotted with anti-TIMP-1, anti-integrin αv, anti-PDGFRβ, anti-vimentin, anti-CD99, anti-CD44 and anti-ZO-1 antibodies, as indicated, by using anti-vinculin antibody as loading control; the histogram indicates the signal intensity of the bands, normalized to the respective anti-vinculin signal. Bars depict means ± SD of three independent experiments. ** *p* < 0.01, **** *p* < 0.0001 relative to MDA-MB-231; unpaired *t*-test. In (**A**,**C**), molecular weights of indicated proteins are reported.

Chemoresistance of TNBC is the main cause of treatment failure and contributes to metastasis, which negatively affects the prognosis of patients [44]. A previous study reported that primary tumor TIMP-1 levels are correlated with resistance to chemotherapy in patients with metastatic breast cancer [45]. To investigate the potential role played by TIMP-1 in TNBC chemoresistance, we first evaluated its expression in two TNBC cell lines, BT-549 and MDA-MB-231, representative of the highly malignant and invasive mesenchymal subtype (MES) [46]. In agreement with previous findings [25], these cells share high TIMP-1 expression compared with the BT-474 epithelial breast cancer cell line (ER+, PR+, HER2 over-expression), with MDA-MB-231 displaying higher levels than BT-549 cells (Figure 1A). Next, we analyzed TIMP-1 expression levels in two MDA-MB-231 derivative cell lines, indicated as Dox-R and Cis-Pt-R, that we generated by chronic treatment of parental cells with Dox and Cis-Pt, respectively [38,39]. Both Dox-R and Cis-Pt-R cell lines, compared with the parental cells, show an increased chemoresistance (Figure 1B) and express higher levels of mesenchymal/stem cell markers as integrin αv [38,47,48,49], PDGFRβ [46,50], vimentin [51], CD99 [52,53] and CD44 [54], and lower levels of ZO-1 epithelial cell marker [55], reflecting their mesenchymal/stem-like phenotype (Figure 1C). Interestingly, TIMP-1 expression in both highly chemoresistant cell lines was significantly higher than in parental MDA-MB-231 cells (Figure 1C).

**Figure 2:**
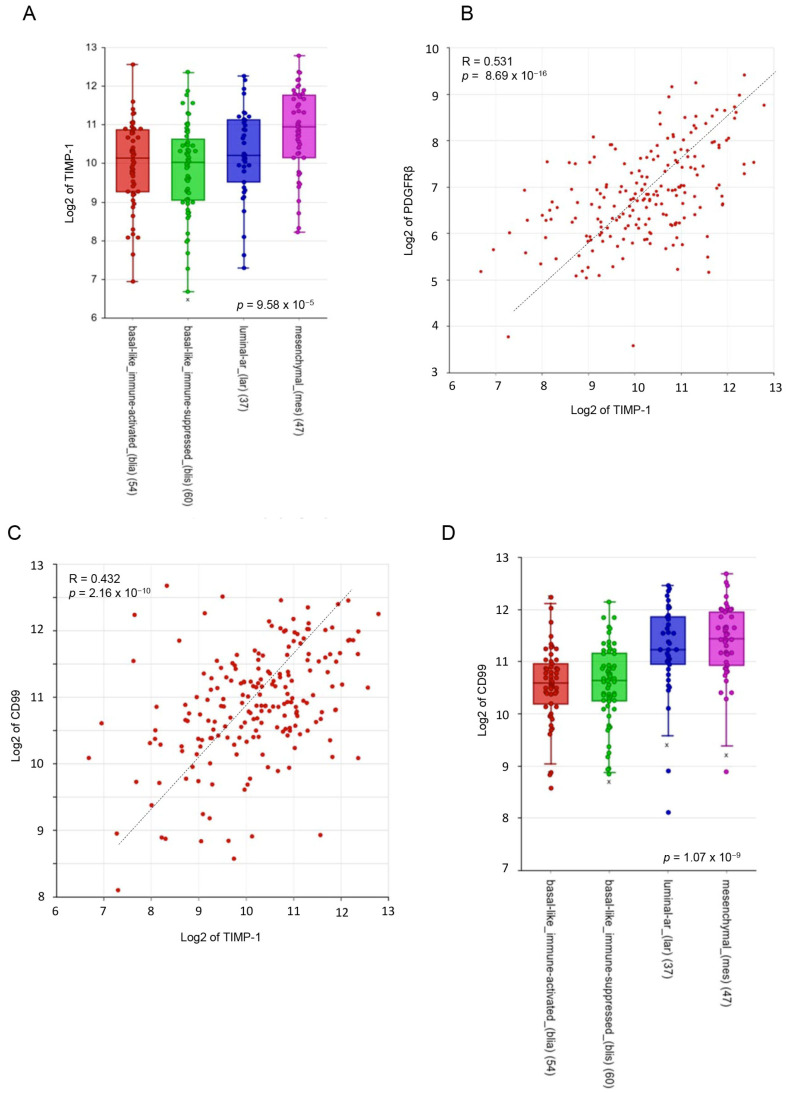

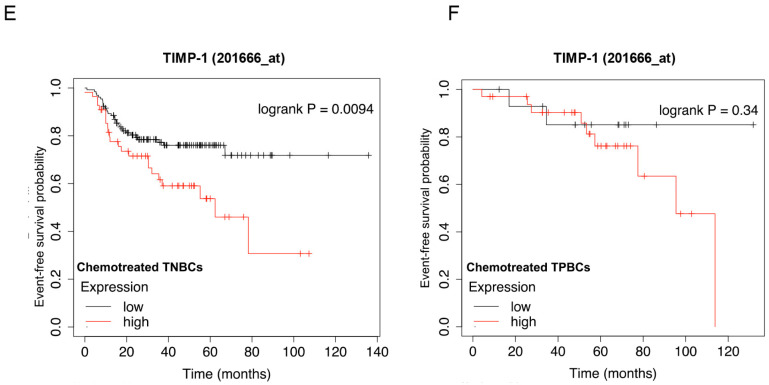
TIMP-1 is enriched in MES TNBC and associated with a worse prognosis in high-grade and chemotreated TNBC patients. (**A**–**D**) Gene expression analysis in a public data set of TNBC (GSE76124) through the R2 Genomic Analysis and Visualization platform (http://r2.amc.nl). (**A**) Box plot comparing TIMP-1 gene expression in different TNBC subtypes. The number of tumors for each subtype are reported in brackets. The data were analyzed by one-way ANOVA through the R2 web platform. (**B**,**C**) XY-dotplots showing the correlation between TIMP-1 (*X*-axis) and either PDGFRβ or CD99 (*Y*-axis) gene expression. (**D**) Box plot comparing CD99 gene expression in different TNBC subtypes. (**E**,**F**) Analysis of the prognostic value of TIMP-1 level in TNBC patients by Event free survival (EFS) Kaplan–Meier curves using a TCGA gene chip mRNA gene array dataset through the KMplot platform (www.kmplot.com). (**E**) Relationship between TIMP-1 expression levels and EFS in 186 TNBC chemotreated patients of high grade; the expression cut-off was set to 9066 in a range from 509 to 29,697, auto-selecting the best cut-off. (**F**) Meta-data analysis of TIMP-1 showed no relationship with EFS in 48 treated TPBC patients of any grade. The expression cut-off was set to 5614. The data were statistically analyzed by one-way analysis of variance (ANOVA) in (**A**,**D**); Pearson’s r coefficient in (**B**,**C**); and log-rank test in (**E**,**F**).

Accordingly, by analyzing TIMP-1 expression in a public dataset (GSE76124) of 198 TNBC samples [41], we found that it is enriched in MES TNBC (Figure 2A) and significantly correlates with the expression of the mesenchymal stem cell marker PDGFRβ (Figure 2B), and CD99 (Figure 2C), a cell surface protein related to chemoresistant mesenchymal cancers [52,56,57] and highly enriched in MES TNBC (Figure 2D).

It has been previously shown, by using a large publicly available clinical breast cancer microarray database, that TIMP-1 expression is associated with poor overall survival (OS) in TNBC patients but not in the entire BC population or in Luminal-A, Luminal-B and HER2+ BC subtypes [25]. However, the prognostic value of TIMP-1 expression in chemotreated TNBC patients has yet to be reported. Thus, we analyzed the prognostic value of TIMP-1 with event-free-survival (EFS) Kaplan–Meier curves using a TCGA RNA gene array dataset obtained from 186 patients with TNBC at third histologic grade, and only treated with chemotherapeutics [42]. In this analysis, higher TIMP-1 mRNA levels were associated with worse prognosis (*p* = 0.0094). Indeed, the 5-year survival was ~50% for the high TIMP-1 group and ~80% for the low TIMP-1 group and upper quartile survival was of 67.00 months for the low-expression group and 17.45 months for the high-expression group (Figure 2E). We also analyzed the correlation of TIMP-1 expression with EFS in treated TPBC (ER+, PR+, HER2 expression) patients of different tumor stages, without finding any significant association with prognosis (*p* = 0.34) (Figure 2F). Thus, high TIMP-1 expression correlates with poor prognosis only in treated TNBC patients.

This, together with our in vitro results indicating that TIMP-1 is significantly upregulated in chemoresistant cells, leaves us to hypothesize that TIMP-1 overexpression confers chemoresistance in TNBC.

### 3.2. TIMP-1 Downregulation Reverses Chemoresistance in TNBC Cells

**Figure 3:**
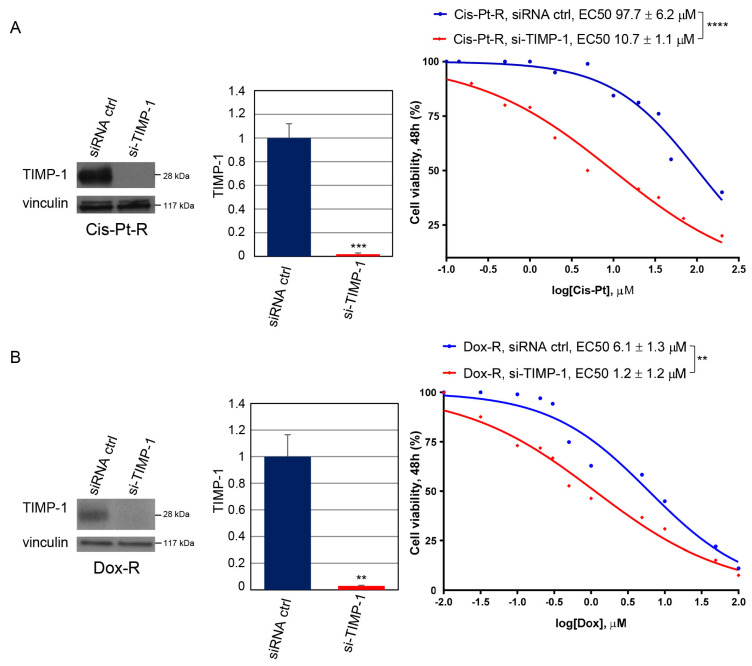
TIMP-1 expression correlates with chemoresistance. Cis-Pt-R (**A**) and Dox-R (**B**) cells were transfected with si-TIMP-1 or siRNA ctrl. Left: at 24 h post-transfection, cells were harvested, and cell lysates prepared and immunoblotted with anti-TIMP-1 antibody. Anti-vinculin antibody was used as a loading control. Molecular weights of indicated proteins are reported. Middle: the histograms indicate the TIMP-1/vinculin ratio of the densitometric signals. Values are shown relative to siRNA ctrl, arbitrarily set to 1. Bars depict means ± SD of three independent experiments. ** *p* < 0.01, *** *p* < 0.001 relative to siRNA ctrl; unpaired *t*-test. Right: cell viability of transfected cells, incubated with increasing concentrations of Cis-Pt (**A**) or Dox (**B**) for 48 h, was determined and expressed as percentage of viable treated cells with respect to untreated controls. EC50 values were determined as reported in the legend to Figure 1B. ** *p* < 0.01, **** *p* < 0.0001; unpaired *t*-test.

To test whether TIMP-1 plays an essential role in chemoresistance, we first checked whether inhibition of TIMP-1 expression using RNAi would affect resistance to Cis-Pt and Dox treatment of Cis-Pt-R and Dox-R cell lines, respectively. To this end, TIMP-1 expression was knocked down by transfecting chemoresistant cells with TIMP-1 targeting siRNA (si-TIMP-1) for 24 h, using scrambled non-targeting siRNA (siRNA ctrl) as a negative control. Then, the transfected cells were treated for 48 h with increasing concentrations of Cis-Pt (ranging from 100 nM to 200 µM) or Dox (ranging from 10 nM to 100 µM) and cell viability was determined using the MTT assay for EC50 values’ calculation. As shown in Figure 3A, Cis-Pt-R cells interfered for TIMP-1 expression (Cis-Pt-R, si-TIMP-1) had a significant higher response to Cis-Pt treatment (*p* < 0.0001) compared to control cells (Cis-Pt-R, siRNA ctrl) expressing high levels of TIMP-1. Indeed, EC50 values for Cis-Pt were 10.7 ± 1.1 µM on TIMP-1 depleted cells and 97.7 ± 6.2 µM on control cells (Figure 3A). Interestingly, cells interfered for TIMP-1 expression had a response to Cis-Pt treatment comparable to that of parental MDA-MB-231 cells (see Figure 1B).

Similarly, TIMP-1 silencing in Dox-R cells (Dox-R, si-TIMP-1) reversed the chemoresistance of the cells (Figure 3B), lowering EC50 values for Dox from 6.1 ± 1.3 µM (control cells) to 1.2 ± 1.2 µM (TIMP-1 interfered cells) (*p* = 0.0087). Again, by interfering for TIMP-1 expression, an EC50 value for Dox comparable to that calculated on parental MDA-MB-231 cells was obtained (see Figure 1B).

### 3.3. TIMP-1 Downregulation Induces Death of Chemoresistant TNBC Cells by Inhibiting AKT Phosphorylation

**Figure 4:**
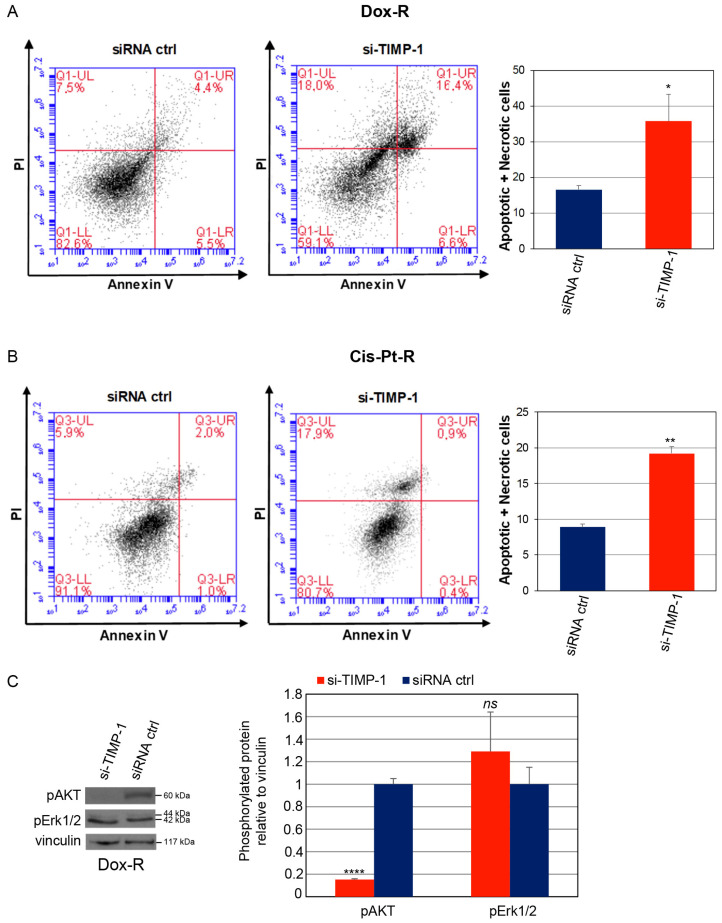

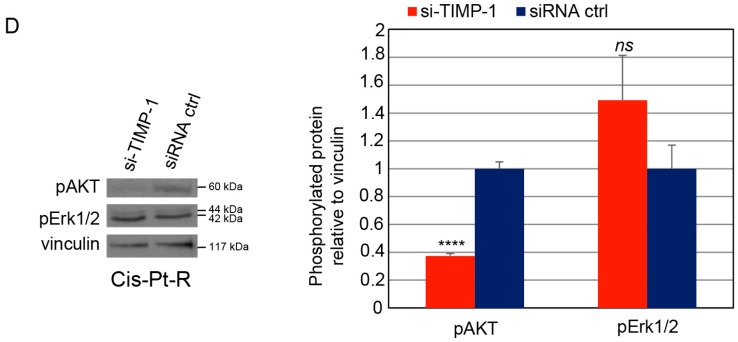
Silencing TIMP-1 expression induces death of chemoresistant TNBC cells. Cell death of Dox-R (**A**) and Cis-Pt-R (**B**) transfected with si-TIMP-1 or siRNA ctrl for 24 h was measured by Annexin-V/PI staining and flow cytometric analysis. Percentage of apoptotic plus necrotic cells is shown on the histogram. (**C**,**D**) Lysates from Dox-R (**C**) and Cis-Pt-R (**D**) cells following 24 h transfection with si-TIMP-1 or siRNA ctrl were immunoblotted with anti-pAKT, anti-pErk1/2 and anti-vinculin antibodies as indicated. Molecular weights of indicated proteins are reported. The histograms indicate the pAKT/vinculin and pErk1/2/vinculin, reported as relative to siRNA ctrl, arbitrarily set to 1. (**A**–**D**) Bars depict means ± SD of three independent experiments. * *p* < 0.05, ** *p* < 0.01, **** *p* < 0.0001 relative to siRNA ctrl; *ns*, not significant; unpaired *t*-test.

In order to evaluate whether TIMP-1 silencing induced cell death per se, both Cis-Pt-R and Dox-R cells were transfected with si-TIMP-1 or siRNA ctrl and analyzed by flow cytometry after Annexin-V/PI dual staining. As shown (Figure 4A,B), interference with TIMP-1 expression significantly increased the proportion of apoptotic plus necrotic cells compared to that of control cells, from ~16.6% to ~35.7% for Dox-R (*p* = 0.0120) and from ~8.9% to ~19.2% for Cis-Pt-R (*p* = 0.0011). Moreover, signaling pathways underlying the mechanisms associated with the reversion of chemoresistance in the presence of TIMP-1 downregulation were investigated. As shown, interfering for TIMP-1 expression in Dox-R (Figure 4C) and Cis-Pt-R (Figure 4D) cell lines caused a strong reduction of phosphorylated AKT (pAKT) without affecting the levels of phosphorylated Erk1/2 (pErk1/2) downstream effector.

### 3.4. Exogenously Added TIMP-1 Confers Chemoresistance to TNBC Cells

**Figure 5:**
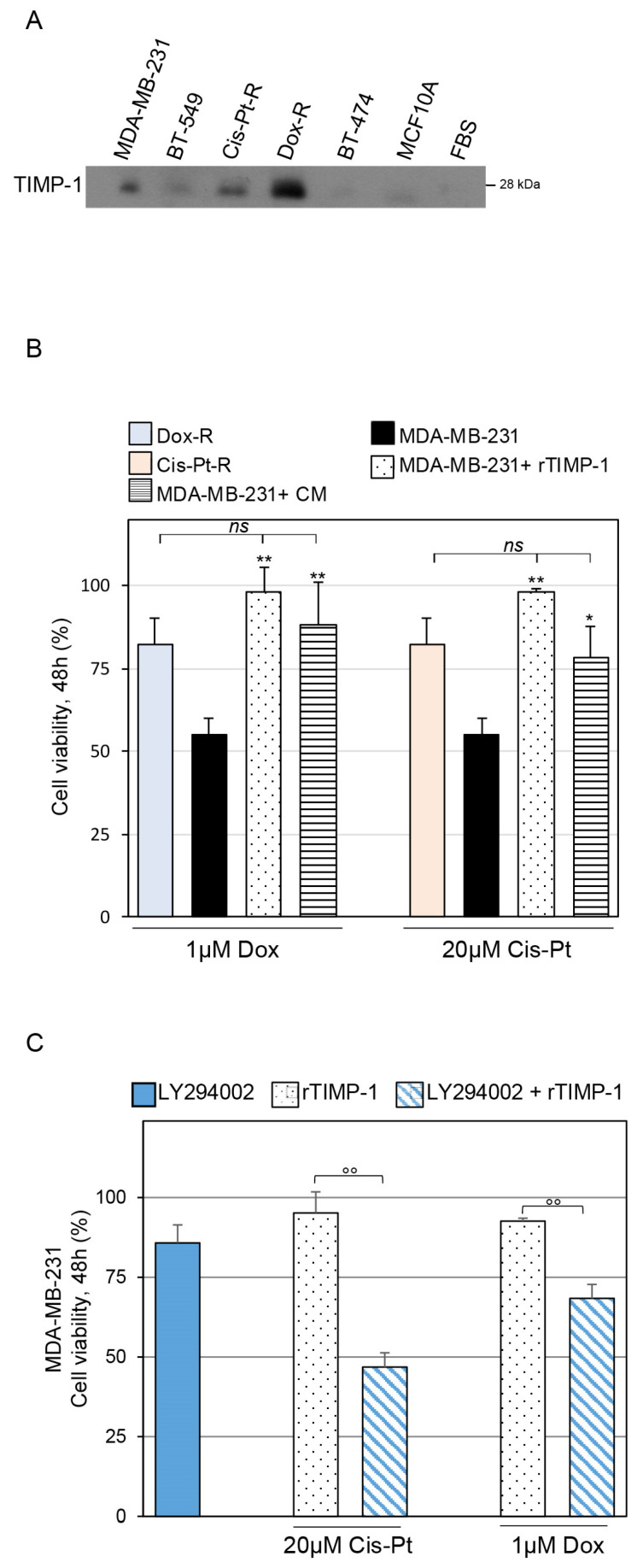
Exogenous TIMP-1 confers chemoresistance to MDA-MB-231 cells. (**A**) The conditioned medium obtained from MDA-MB-231, BT-549, Cis-Pt-R, Dox-R, BT-474 and MCF10A cells was analyzed by immunoblotting using anti-TIMP-1 antibody. FBS was used to exclude the presence of TIMP-1 in the serum supplementing the culture medium. (**B**) MDA-MB-231 cells were left untreated or treated for 48 h with 1 µM Dox or 20 µM Cis-Pt alone or in combination with either recombinant TIMP-1 (rTIMP-1) or TIMP-1-enriched CM obtained from Dox-R cells. Cis-Pt-R and Dox-R cells left untreated or treated with 20 µM Cis-Pt or 1 µM Dox, respectively, were used as chemoresistant reference cells. (**C**) MDA-MB-231 cells were treated for 48 h with rTIMP-1 plus 1 µM Dox or 20 µM Cis-Pt, in the presence or in the absence of 10 µM LY294002. In (**B**,**C**), cell viability was analyzed and expressed as percent of viable treated cells with respect to untreated cells. Bars depict means ± SD of three independent experiments. * *p* < 0.05; ** *p* < 0.01, relative to treated MDA-MB-231 cells; *ns*, not significant; °° *p* < 0.01, one-way ANOVA followed by Tukey’s multiple comparison test.

To gain further insight into the involvement of TIMP-1 in chemoresistance, we investigated whether exposure of parental MDA-MB-231 cells to exogenously added TIMP-1 confers resistance to Cis-Pt and Dox treatment. To this aim, we first analyzed by immunoblotting the level of TIMP-1 in the conditioned media (CM) obtained from MDA-MB-231, BT-549, Cis-Pt-R, Dox-R, BT-474 and MCF10A cells, left in culture for 72 h. As shown (Figure 5A), TIMP-1 was detected in the CM obtained from all TNBC cell lines, particularly in Dox-R cells, whereas its levels were almost undetectable in the medium obtained from both TPBC BT-474 and non-tumorigenic MCF10A cells. Next, we performed MTT assays to compare the chemotherapeutic response between parental MDA-MB-231 cells, treated with TIMP-1, either in a recombinant soluble form or released into the CM obtained from Dox-R cells, and chemoresistant MDA-MB-231-derivative cell lines (Figure 5B). Thus, we treated MDA-MB-231 cells with 1 µM Dox or 20 µM Cis-Pt, concentration values around the EC50 on parental MDA-MB-231 cells (Figure 1B and [38]), alone or in combination with either recombinant TIMP-1 (25 ng/mL) or CM obtained from Dox-R cells and, at 48 h treatment, evaluated their viability. Importantly, both the CM and recombinant TIMP-1 strongly increased the resistance of MDA-MB-231 cells to both Cis-Pt and Dox, leading to cell viability values comparable to those of chemoresistant Cis-Pt-R and Dox-R cells, respectively, treated with the same concentrations of chemotherapeutics (Figure 5B). Furthermore, recombinant TIMP-1 was unable to induce chemoresistance in MDA-MB-231 parental cells treated with LY294002 inhibitor (Figure 5C), thus suggesting that TIMP-1 could mediate chemoresistance by promoting the PI3K/AKT pathway.

### 3.5. TIMP-1 Associates with the Cell Surface TNBC by Binding to CD63

**Figure 6:**
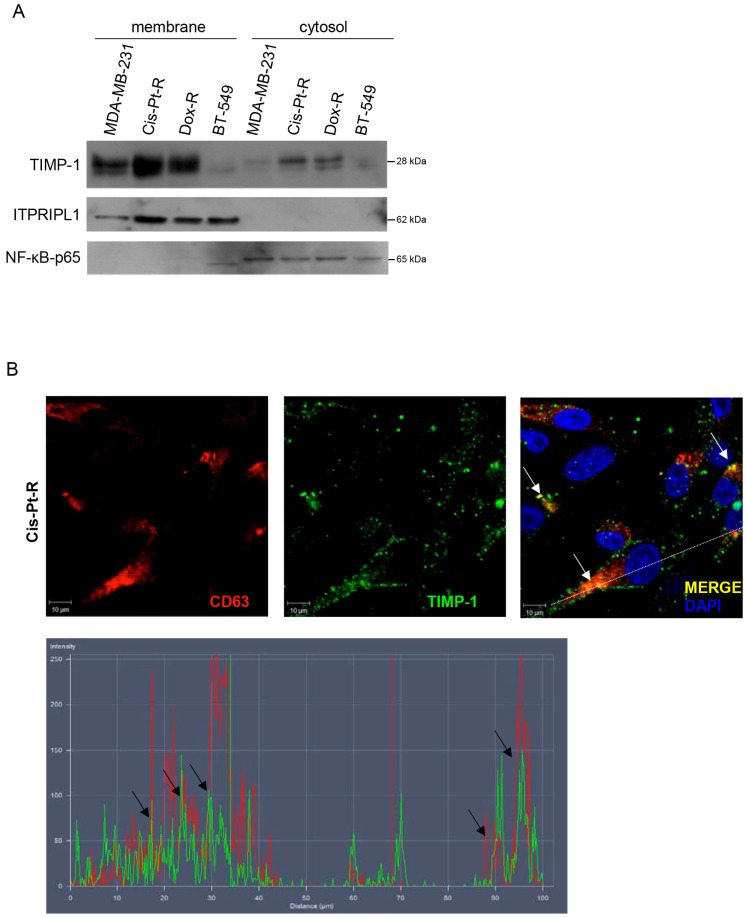

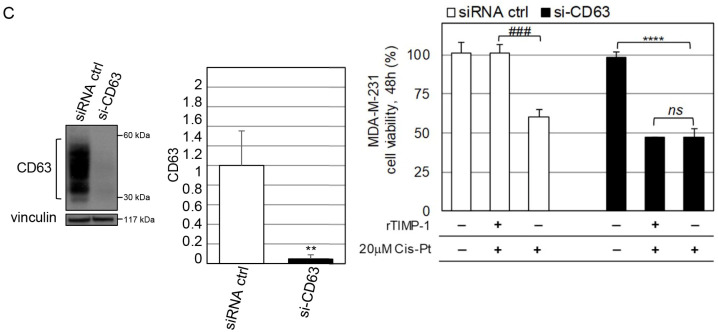
TIMP-1 binds to CD63 cell surface receptor. (**A**) Cell membrane and cytosolic fractions from the indicated cell lines were analyzed by immunoblotting using anti-TIMP-1 antibody. The cell-membrane receptor ITPRIPL1 and the cytosolic protein NF-kB-p65 were used as controls for the extraction procedure. (**B**) Confocal microscopic analysis of Cis-Pt-R cells co-stained with anti-TIMP-1 (green) and anti-CD63 (red) antibodies. Nuclei are visualized in blue. Magnification 63×, 1.0× digital zoom, scale bar = 10 μm. The merged image shows the overlay (yellow) of the two channels; white arrows indicate some points of colocalization between CD63 and TIMP-1. The representative section indicated by the dashed line was used for fluorescence intensity profiling analysis. Intensity peaks representing the red (CD63) and green (TIMP-1) emission wavelengths indicate areas of colocalization (black arrows). (**C**) MDA-MB-231 cells were transfected with si-CD63 or siRNA ctrl. Left: at 24 h post-transfection, cells were harvested, and cell lysates prepared and immunoblotted with anti-CD63 antibody. Anti-vinculin antibody was used as a loading control. Molecular weights of indicated proteins are reported. Middle: the histogram indicates the CD63/vinculin ratio of the densitometric signals. Values are shown relative to siRNA ctrl, arbitrarily set to 1. Bars depict means ± SD of three independent experiments. ** *p* < 0.01 relative to siRNA ctrl; unpaired *t*-test. Right: at 24 h post-transfection, cells were treated with 20 µM Cis-Pt alone or in combination with rTIMP-1. Cell viability was analyzed and expressed as percentage of viable treated cells compared to untreated cells. Bars depict means ± SD of three independent experiments. **** *p* < 0.0001, ### *p* < 0.001; *ns*, not significant; one-way ANOVA followed by Tukey’s multiple comparison test.

It has been shown that secreted TIMP-1 reassociates with the cell plasma membrane, through CD63 receptor partner, to exert its MMP-independent activities in various cell types [34,58]. Thus, as a first step to evaluate whether TIMP-1 associates with TNBC cell surface to promote chemoresistance, membrane proteins of MDA-MB-231, Cis-Pt-R, Dox-R cells and BT-549 cells were separated from the intracellular protein fraction and TIMP-1 levels detected by immunoblotting. As shown in Figure 6A, TIMP-1 was present in both cell compartments but highly enriched in cell membranes. ITPRIPL1 receptor and NF-kB-p65 were used as markers of cell-surface and cytosolic compartments (www.proteinatlas.org), respectively. Next, we verified the expression of both CD63 and integrin β1 in MDA-MB-231, Cis-Pt-R, Dox-R and BT-549 cells by confocal microscopy (Figure S1A) and immunoblotting (Figure S1B) and their interaction in Cis-Pt-R cells, chosen as a model system, by immunoprecipitation analysis (Figure S1C). Finally, TIMP-1 binding to CD63 was verified by confocal microscopy analyses of cells co-stained with specific CD63 and TIMP-1 antibodies (Figure 6B).

These results suggest that CD63 acts as a partner receptor for TIMP-1 to promote its induction of chemoresistance. To validate this hypothesis, parental MDA-MB-231 cells were interfered for CD63 expression, treated with recombinant TIMP-1, and tested for TIMP-1-induced chemoresistance. As shown (Figure 6C), in the absence of CD63 expression, Cis-Pt-induced inhibition of cell viability was not affected by TIMP-1 treatment.

All these data indicate that TIMP-1 plays a key role in TNBC resistance to Cis-Pt and Dox through binding to CD63.

## 4. Discussion

TNBC is a highly heterogeneous tumor comprising subtypes that differ not only in histopathology and gene expression, but also in prognosis and response to therapies [41,59]. Because of the lack of actionable biomarkers for managing each specific subtype, therapy for TNBC still remains inadequate, with only four targeted therapies (PARP inhibitors, immunomodulatory Atezolizumab and Pembrolizumab monoclonal antibodies, and Sacituzumab Govitecan antibody–drug conjugate) applicable to a restrict number of TNBC patients and always in combination with a chemotherapeutic regimen [60]. Thus, most patients with TNBC are still treated exclusively with chemotherapy. Tumor size, lymph node status, grade and the presence or absence of medical co-morbidities usually determine the type of regimen to use [61]. TNBC chemotherapy generally consists of a combined regimen of taxanes (paclitaxel, docetaxel), anthracyclines (doxorubicin, epirubicin), alkylating agents (cyclophosphamide), platinum and fluorouracil [62,63,64].

While anthracycline-based chemotherapy, including Dox, is among the current standard of systemic TNBC treatment [62], Cis-Pt does not represent the standard of care, even though there is a considerable interest for its use, especially for BRCA1/2 mutation carriers, who represent approximately 20% of TNBC patients [64,65]. Moreover, given the evidence that PARPis are active in patients previously treated with platins, first-line therapy with carboplatin or Cis-Pt as single agents is considered an appropriate treatment option [64]. Several trials of platinum-based chemotherapies are currently underway both as neoadjuvant regimens and in metastatic settings [43,66,67].

Unfortunately, primary TNBC carries high chemosensitivity but higher risk of early relapse than other breast cancers, a situation known as the triple-negative paradox [68]. It has been shown that tumor cells surviving chemotherapy demonstrate a shift towards a more mesenchymal and stem-like phenotype that supports chemoresistance, tumor relapse and metastasis promotion. Moreover, at the metastatic site, host responses to therapy activate inflammatory pathways, leading to the formation of a favorable microenvironment able to receive cancer cells [69]. As such, continuous efforts are devoted to finding more effective chemotherapeutic regimens for responders and non-responders and to understanding the mechanisms of chemoresistance in order to improve the treatment of patients with TNBC.

Here, in agreement with previous findings [70,71], we found that TNBC cells grown under the selective pressure of Cis-Pt or Dox acquire chemoresistance and a stem cell-like/EMT phenotype. Indeed, chemoresistant cell lines share a higher level of proteins preferentially expressed by cancer cells with stem-like characteristics and/or that have undergone EMT transition, such as CD44 [54], PDGFRβ [46,50], CD99 [52,53], vimentin [51], integrin αv and integrin β1 [47,48], and lower levels of ZO-1 chosen as epithelial marker [55], compared to parental MDA-MB-231 cells. Furthermore, in agreement with the evidence that chemotherapy induces the enrichment of programmed death-ligand 1 (PD-L1)-positive immune-evasive TNBC cells [72], we previously showed a significant increase, in both Cis-Pt-R and Dox-R chemoresistant cell lines, of PD-L1 expression [38], which has an established tumor-intrinsic signaling promoting EMT in TNBC cells [73,74,75,76].

Importantly, we show that both cytosolic and secreted levels of TIMP-1 were significantly increased in either Cis-Pt-R and Dox-R chemoresistant cell lines with respect to parental cells. Moreover, interrogation of the TCGA database allowed us to confirm that the expression of TIMP-1 is correlated with a high rate of relapses and mortality in chemotreated TNBC patients and is higher in mesenchymal-stem-like TNBC with respect to other subtypes.

It is known that in several human tumors, TIMP-1, once released from cancer or stromal cells, associates with CD63 at the cell surface, exerting its oncogenic role [16]. Also, evidence has been reported that indicates TIMP-1 as a regulator of essential stem cell functions. Indeed, RNA interference of TIMP-1 in human mesenchymal stem cells (hMSCs) has revealed that endogenous TIMP-1, once secreted by hMSCs, colocalizes with CD63 and acts as a suppressor of osteogenic differentiation through negative modulation of the Wnt/β-catenin signaling [58]. Furthermore, in glioblastoma, the co-expression of TIMP-1 and stem cell markers, as well as the expression of CD63, has suggested a role for TIMP-1 and CD63 in cancer cell stemness [77].

Some recent studies on established cancer cell lines belonging to NSCLC [36] and ovarian cancer [37] have also suggested a potential role for TIMP-1 in Cis-Pt resistance. Moreover, by analyzing the proteome and phosphoproteome differences of MCF-7 breast cancer cells expressing high or low levels of TIMP-1, the upregulation and hyperphosphorylation of proteins that are directly or indirectly associated with drug resistance were detected in TIMP-1 high-expressing cells [78].

However, the role of TIMP-1 as a mediator of chemoresistance in TNBC still remains unexplored. Our results, for the first time, suggest a role for TIMP-1/CD63 axis in TNBC cells’ chemoresistance. In fact, silencing of TIMP-1 in chemoresistant Cis-Pt-R and Dox-R cells reversed cell resistance to Cis-Pt and Dox, respectively, whereas treating parental TNBC cells with exogenously added TIMP-1, either in a recombinant form or enriched in conditioned media harvested from chemoresistant cells, conferred resistance to Cis-Pt and Dox. Moreover, TNBC cells interfered for CD63 expression did not respond to TIMP-1 treatment anymore.

DNA damage through chemotherapy has been shown to induce cancer cell exosome secretion, which has a critical role in tumor progression, including the transfer of chemoresistance to surrounding cells [79,80]. Moreover, colorectal-cancer-derived and TIMP-1-enriched extracellular vesicles were recently found to upregulate TIMP-1 levels in recipient liver fibroblasts and induce ECM remodeling, which could be a precursor event in the establishment of a metastatic niche in the liver [81]. Therefore, it is plausible to envisage that tumor cell release of CD63 and TIMP-1 via exosomes might result in the horizontal transfer of malignant traits to recipient cells, thereby promoting tumor progression and resistance to therapy.

It has been reported that tetraspanins, including CD63, promote proliferation, migration and chemoresistance via activating PI3K/AKT signaling [82], and that TIMP-1 mediates apoptosis by activating cell survival signaling pathways involving PI3K/AKT [28]. Interestingly, we found that TIMP-1 silencing in chemoresistant Cis-Pt-R and Dox-R cells inhibits AKT phosphorylation, and that LY294002, a specific inhibitor of the PI3K/AKT pathway, makes soluble TIMP-1 unable to induce chemoresistance in MDA-MB-231 parental cells, thus suggesting that TIMP-1 could mediate chemoresistance by promoting this pathway. Nevertheless, we cannot exclude the occurrence of mechanisms based on the anti-proteolytic activity of TIMP-1, which could also create an additional layer of complexity. In this regard, evidence shows that TIMP-1, by suppressing ADAM-10, a metalloproteinase responsible for the shedding of the c-Met receptor on the cell surface, promotes liver metastasis by induction of the hepatocyte growth factor-c-Met axis [83]. Similarly, TIMP-1 could act as an inhibitor of proteinases responsible for the shedding of receptors that in turn function as decoys and withdraw available pro-survival ligands.

Overall, drug resistance in cancer cells is a complex and multifactorial process, which includes reduced drug uptake, increased drug efflux, enhanced DNA repair, altered drug metabolism and changes in apoptotic pathways [84]. Different mechanisms can act together or independently to confer resistance to Cis-Pt and Dox, which both exert their anti-cancer effects by damaging DNA and inducing cancer cell death by triggering apoptotic pathways [70,84]. Resistance can develop through alterations in these pathways, including AKT activation, leading to decreased susceptibility to apoptosis [84]. Therefore, TIMP-1 could induce Cis-Pt and Dox drug resistance by activating the AKT signaling pathway. The mechanisms through which TIMP-1 can activate AKT are multiple, including RTK activation, PTEN inhibition, integrin signaling and EMT induction [16,17,20]. Further studies are needed to fully understand all molecular details of this interaction and its significance in drug resistance.

Taken together, our results encourage in vivo investigation to confirm the role of TIMP-1 in mediating chemoresistance of TNBC and suggest that hampering TIMP-1/CD63 complex with specific inhibitors may provide a viable approach to enhance the efficacy of chemotherapy in TNBC.

## 5. Conclusions

In summary, the present study identified TIMP-1 overexpression in chemoresistant TNBC cell lines and tumor samples and demonstrated that knockdown of its expression reverses cell resistance to both cisplatin and doxorubicin, thereby inducing cell death, possibly through inhibition of the AKT pathway. Binding of TIMP-1 to the CD63 receptor is critical for this action. These results suggest that TIMP-1 signaling is a promising actionable target for novel therapeutic approaches against chemoresistant TNBC. However, further explorations in TNBC murine models will allow us to confirm the role of TIMP-1 in TNBC chemoresistance.

## Data Availability

Not applicable.

## Supplementary Materials

The following supporting information can be downloaded at: https://www.mdpi.com/article/10.3390/cells12131809/s1, Figure S1: CD63 and integrin β1 expression in TNBC cells.
