# Supplementary material for: Tissue Inhibitor of Metalloproteinases-1 Overexpression Mediates Chemoresistance in Triple-Negative Breast Cancer Cells
Source: Cells. 2023 Jul 7;12(13):1809. doi: 10.3390/cells12131809 (PMC10340747; doi:10.3390/cells12131809)
Supplement: Supplementary file 1 [file cells-12-01809-s001.zip › cells-2467037-supplementary.pdf]

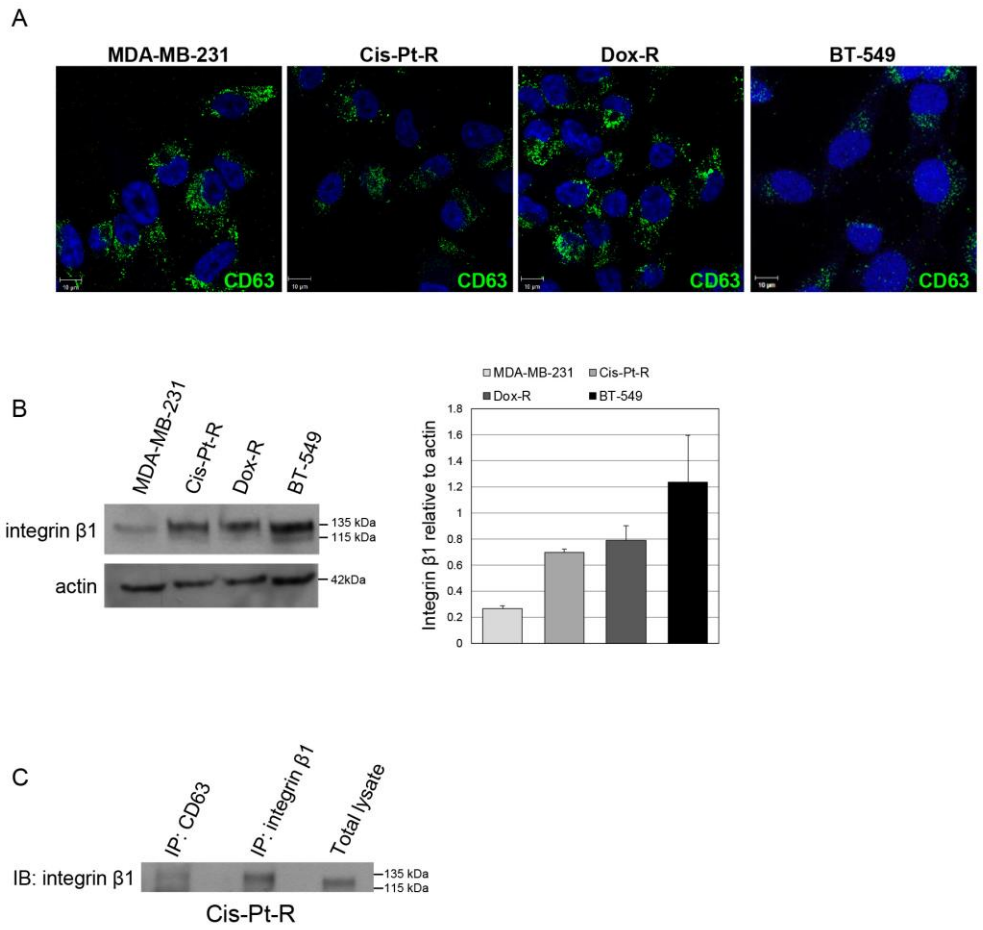

Figure S1. CD63 and integrin β1 expression in TNBC cells. (A) MDA-MB-231, Cis-Pt-R, Dox-R and BT-549 cells were fixed and labelled with anti-CD63 antibody (green). Nuclei are visualized in blue. Magnification 63×, 1.0× digital zoom, scale bar = 10 μm. All digital images were captured at the same setting to allow direct comparison of staining patterns. (B) Lysates from MDA-MB-231, Cis-Pt-R, Dox-R and BT-549 cells were immunoblotted with anti-integrin β1 antibody, by using anti-actin antibody as loading internal control. Molecular weights of indicated proteins are reported. The histogram indicates the integrin β1/actin ratio. Bars depict means ± SD of three independent experiments. (C) Anti-CD63 or anti-integrin β1 immunoprecipitates (IP) from Cis-Pt-R cell lysates were subjected to immunoblot using anti-integrin β1 antibody (IB). Total lysate before immunoprecipitation served as a control.
